# Inactivation of *Salmonella* enteritidis in liquid egg yolk and egg white using bacteriophage cocktails

**DOI:** 10.1016/j.crfs.2024.100703

**Published:** 2024-02-24

**Authors:** Jiangning He, Catherine W.Y. Wong, Danielle M. Schultze, Siyun Wang

**Affiliations:** Food, Nutrition and Health, The University of British Columbia, 2205 East Mall, Vancouver, BC, V6R 1Z4, Canada

**Keywords:** Bacteriophages, *Salmonella* enteritidis, Liquid eggs

## Abstract

Salmonella Enteritidis (SE) is a significant global cause of foodborne illness, often linked to egg contamination. This study evaluated the inhibitory effects of eight bacteriophages (phages) against three SE strains isolated from poultry environments. The most effective phages were selected to formulate different phage cocktails, to enhance the efficacy and prolong inhibition. Four phage cocktails were tested at a multiplicity of infection (MOI) of 100 in tryptic soy broth (TSB), and at MOIs of 100 and 1000 in liquid egg white (EW) and egg yolk (EY) with storage at 8 °C for up to 30 days (d). The effectiveness of the phage cocktails varied significantly among bacterial strains, yet all demonstrated significant reductions compared to the positive control in liquid culture (P < 0.05). Similarly, the tested SE strains in both EW and EY showed significant reductions with phage treatments (P < 0.005), although the effectiveness was influenced by the MOI and medium composition. Treating EY proved to be more challenging, with lower magnitudes of reduction and longer treatment durations required, compared to EW. Reductions ranged from 1 to greater than 4 log CFU/mL in EW and EY after 30 d, with consistently higher reductions achieved at MOI 1000. Phage titers decreased initially, but remained stable following SE inoculation in broth and liquid eggs at 8 °C, indicating that lysis from without mechanisms may have contributed to the inhibitory effect. Notably, phages exhibited stronger attachment to SE in EW, which can be attributed to be less viscous nature of EW compared to EY. This study demonstrated that phage applications in both EW and EY effectively reduced SE counts at 8 °C, with no regrowth during long-term storage. These findings contribute to the development of biocontrol methods that enhance food safety and reduce foodborne outbreaks associated with contaminated egg products.

## Highlights

• Phages reduced *Salmonella* by 1–7 log CFU/mL in egg white and yolk after 30 days.
• MOI 1000 achieved <3 log CFU/mL greater reductions compared to MOI 100.
• Phage cocktail SF1+SE18 most effectively reduced populations of *Salmonella* S35 and S47.
• Phage cocktail SS4+SE18 most effectively reduced populations of *Salmonella* S26.

## Graphical abstract

**Figure:**
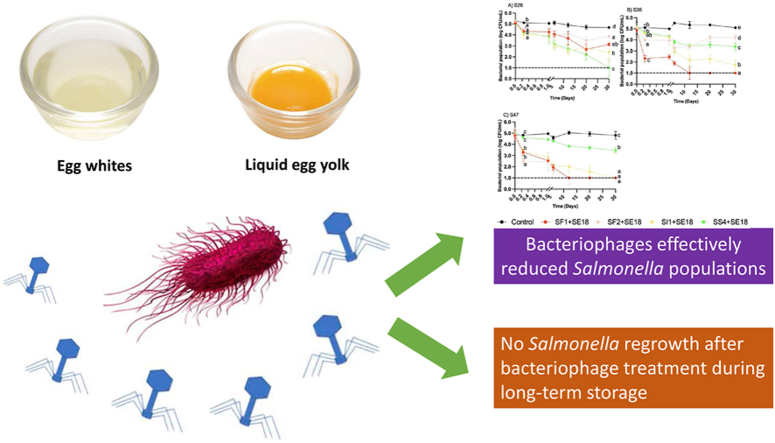

## 1 Introduction

*Salmonella* Enteritidis (*Salmonella enterica* subspecies *enterica* serovar Enteritidis, or SE) is a Gram-negative rod-shaped bacterium, and one of the most frequently reported *Salmonella enterica* serovars (Ferrari et al., 2019). In Canada, SE isolates have increased dramatically since 2003 making it the most prevalent serotype associated with human salmonellosis (Health Canada, 2012). Epidemiological data in Europe and the United States (US) have shown that eggs are most commonly associated with increased illness in humans, and SE accounted for 85% of all cases of human salmonellosis in Europe (Cardoso et al., 2021). In Australia in 2011, 45% of all salmonellosis outbreaks were linked to eggs or egg-related products (Keerthirathne et al., 2016). Approximately 79,000 cases of foodborne illness and 30 deaths each year were caused by the consumption of eggs contaminated with SE in the US (FDA, 2009). It is generally agreed that the prevalence of *Salmonella* in commercial table eggs is low in most developed countries (Martelli and Davies, 2012), and Ebel and Schlosser (2000) estimated that one in every 20,000 eggs annually produced in the US was positive for *Salmonella* (0.005%). Several outbreaks have been linked to food products containing raw and/or unpasteurized eggs such as salad dressing, mayonnaise, mousse, and tiramisu (EFSA & ECDC, 2010; Howard et al., 2012). Other extrinsic factors such as temperature abuse, the level of undercooking, contamination of ready-to-eat foods and cross-contamination may affect *Salmonella* contamination (Oscar, 2020).

SE has been isolated from all sites in the hen reproductive tract, thus making contamination possible in both egg yolk (EY) and egg white (EW) (Whiley and Ross, 2015). Some studies reported that EW contamination is more common (Gast and Beard, 1990; Humphrey et al., 1991), while other studies have shown that EY contamination is more frequent due to the high nutrient content (Gast and Holt, 2001; Gast et al., 2007). Egg contamination may occur at different stages of formation, processing, packaging and shipping, and poses a serious health threat to the public as *Salmonella* growth does not result in visible changes to the color, smell, or consistency of the egg contents (Humphrey and Whitehead, 1993; Whiley and Ross, 2015).

Due to the emergence of multi-drug resistant pathogens, researchers are increasingly looking to bacteriophages (phages) as an alternative mitigation strategy (Endersen et al., 2014). Phages show promise as biocontrol agents for food safety due to their high specificity and ability to self-replicate without impacting beneficial microflora and human cells (Babickova and Gardlik, 2015). Phages have been approved for food safety applications by the Food and Drug Administration (FDA), and Salmonelex, EcoShield, SalmoFresh, and SalmoProTM are examples of phage-based biocontrol agents that have been approved for use in poultry, red meat, eggs, fish, shellfish, and post-harvest fruits and vegetables to control *Salmonella* (US Food and Drug Administration, 2015). Thus, phages are a viable alternative to traditional antimicrobials for ensuring food safety.

Numerous studies have investigated phage treatment applications in eggs; however, the majority used shelled eggs as opposed to liquid eggs. For example, Spricigo et al. (2013) reported a 0.9 log CFU/cm^2^ reduction of SE and *S.* Typhimurium after 2 h (h) on whole eggs dipped into bacterial suspensions containing 10^7^ CFU/mL and sprayed with a phage cocktail containing 10^10^ plaque-forming units (PFU)/mL. Sonalika et al. (2020) conducted a similar study in which bacterially contaminated whole eggs were immersed in a solution of phage PSE5, and a 3-log reduction of SE was observed after 30 min (min). Zhang et al. (2021) found that phage Pu20 effectively reduced two strains of multi-drug resistant *Salmonella* in liquid EW and EY, with a stronger antibacterial effect observed in EW stored at 4 °C and 25 °C compared to EY. Similarly, Rahim et al. (2023) demonstrated the effectiveness of phage Rostam in reducing SE growth in both liquid whole eggs and on eggshells. Bacterial growth was reduced below the limit of detection (LOD) at 4 °C in liquid whole eggs within 24 h, while reductions of 2.3 log CFU/egg were achieved on eggshells after 24 h.

The physical characteristics of the food product intended for phage application is an important factor in determining effectiveness. Liquid eggs are more suitable for phage treatment since phages have a higher chance of encountering their host bacteria in liquid environments due to liquid flow and active bacterial motility (Murray and Jackson, 1992). In more viscous media or solid surfaces, increasing the phage concentration may be one solution to overcome challenges relating to motility (Seo et al., 2016). Increasing phage concentration can produce faster reductions of bacteria, as Seo et al. (2016) reported that 10^4^ PFU/cm^2^ of phages completely inhibited *E. coli* O157:H7 after 72 h, whereas similar inhibition with 10^5^ PFU/cm^2^ was achieved after 8 h. The objective of this study was to investigate the efficacy of different phage cocktail formulations in reducing SE in liquid EW and EY.

## 2 Materials and methods

### 2.1 Salmonella strains and culture conditions

The *Salmonella* strains used in this study, *S.* Enteritidis S26, *S.* Enteritidis S35, and *S.* Enteritidis S47, were isolated from various environmental and diagnostic poultry samples by the British Columbia (BC) Ministry of Agriculture (Brenner et al., 2020). The *Salmonella* strains were prepared for long-term storage at −80 °C in Tryptic Soy Broth (TSB) supplemented with 20–25% glycerol. Working stocks were prepared by streaking glycerol stocks onto Tryptic Soy Agar (TSA), and incubating aerobically for 18 ± 2 h at 37 °C followed by storage at 4 °C for up to three weeks.

### 2.2 Phage propagation

The eight phages used in this study were isolated from various sources, including irrigation water, sediment, cattle feces, and sewage effluent (Fong et al., 2017). Phages were propagated to increase their titers to at least 1 × 10^8^ PFU/mL. The propagation of phages was carried out using the soft-agar overlay method, using the isolation host as the propagation host. Phages SI1, SS4, SF1 and SF2 were propagated using *S*. Enteritidis FSL S5-483, while phages SE14, SE15, and SE18 were propagated using *S*. Typhimurium FSL S5-536, and phage SS9 was propagated using *S*. Saintpaul FSL S5-649. Briefly, the phage suspension was mixed with a 1:10 dilution of the host bacterium in 0.7% molten agar, and the mixture was poured onto TSA plates. The plates were incubated overnight at 37 °C, and lysis was observed by the formation of plaques. The plaques were collected by scraping them into 5–10 mL of salt-magnesium (SM) buffer (0.1 M NaCl, 0.05 M Tris-HCl, and 0.01 M MgSO4, pH 7.5) in a 50 mL conical centrifuge tube using a sterile plate spreader. The tube was incubated at room temperature overnight to allow the phage particles to release from the agar into the SM buffer. Subsequently, the samples were centrifuged at 4, 696×*g* for 20 min at 4 °C. The supernatant was then filtered through a 0.45 μm syringe filter to obtain a purified phage sample, which was stored at 4 °C for future experiments. Titers of purified phage stocks were determined by the double-layer agar overlay assay. Subsequent enumerations were conducted one day (d) prior to the experimental start date.

### 2.3 In-vitro infectivity assays of individual phages and phage cocktails

Phages that exhibited high propagation ability and strong lytic activity against the selected bacterial strains were evaluated at a multiplicity of infection (MOI) 100. Firstly, 1 mL of overnight bacterial culture was transferred to 9 mL of TSB and incubated aerobically at 37 °C with agitation until the optical density at 600 nm (OD600) reached 0.4 (5 × 10^6^ CFU/mL in log phase). Subsequently, 100 μL of the bacterial culture was added in triplicate to a 96-well plate, along with 100 μL of diluted phage to achieve a MOI of 100. A plate reader (SpectraMax M2; Molecular Devices, Sunnyvale, CA) was used to measure the OD600 every 30 min for 72 h at 25 °C to assess bacterial cell abundance. Each phage was tested in triplicate against each SE isolate. This assay was repeated using six different phage cocktails formulated by mixing the phages in a 1:1 vol ratio. Positive control wells contained SE without phages, while negative control wells contained 200 μL of TSB.

Phage cocktails were designed based on our previous findings of phage combinations with demonstrated efficacy against *Salmonella* in broth-based assays (Brenner et al., 2020) as well as individual phage infectivity assays from this study. For each phage cocktail treatment, 1 mL of the 1 × 10^6^ CFU/mL SE strain was inoculated into 8 mL of TSB, and 1 mL of the respective phage cocktail at a concentration of 1 × 10^8^ PFU/mL was added, resulting in a MOI of 100. The positive control consisted of 1 mL of the 1 × 10^6^ CFU/mL SE strain inoculated into 9 mL of TSB, while the negative control contained 0.1% buffered peptone water (BPW) instead of the phage cocktail. Samples were incubated at 8 °C and enumerated for both phage titer and SE concentration after 0 and 6 h, and 1, 6, 12, 20, and 30 d. Three independent replicate experiments were conducted.

### 2.4 Single-step growth curves

Single-step growth curves were constructed to determine the phage life cycles following the method described by Fong et al. (2017) with minor modifications. Cultures of *S.* Enteritidis FSL S5-483 were grown for 16 h in TSB at 37 °C and 170 rpm. An aliquot of 1 mL of culture was added to 9 mL of fresh TSB and incubated at 37 °C until the OD600 reached 1.0 (10^9^ CFU/mL in stationary phase). Next, 1 mL of the bacterial culture was transferred to a 1.7 mL microcentrifuge tube, and phages were added at a MOI of 0.01, or approximately 10^7^ PFU/mL. After a 5 min adsorption period at room temperature, the mixture was centrifuged at 4000×*g* and 4 °C to remove unabsorbed phage particles. The pellet was resuspended in 1 mL of fresh TSB and incubated at room temperature with gentle agitation. Subsequently, 50 μL aliquots were collected every 5 or 10 min for 120 min, serially diluted in SM buffer, and spotted in duplicate on a 0.7% TSA overlay with a host agar lawn of *S.* Enteritidis FSL S5-483 grown for 16 h for titer determination. Plates were incubated at 37 °C for 18 ± 2 h to visualize plaques. This experiment was independently conducted in triplicates for each phage. The burst size of a single phage isolate was calculated (El-Dougdoug et al., 2019) using the formula:

Burst size = (phage titer at the end of the burst cycle− initial phage titer) /(initial phage titer)

Where phage titer was measured as PFU/mL.

### 2.5 Liquid egg preparation and inoculation

Overnight bacterial cultures were obtained by inoculating the bacterial strains to 5 mL of TSB and incubating them at 37 °C with agitation (175 rpm) for approximately 18 ± 2 h. The overnight cultures were then centrifuged at 4000×*g* for 5 min, and the resulting pellets were washed twice with 5 mL of sterile BPW. The pellets were resuspended in 5 mL of BPW, and the bacterial concentration was determined by measuring the optical density at 600 nm (OD600) (OD600 = 1 is approximately 1 × 10^9^ CFU/mL in log phase). The inoculum was serially diluted to a concentration of 1 × 10^6^ CFU/mL. Phage stock samples, stored at 4 °C, were diluted in SM buffer to achieve concentrations of 1 × 10^8^ PFU/mL and 1 × 10^9^ PFU/mL, corresponding to MOIs of 100 and 1,000, respectively.

Whole eggs were purchased from a local grocery store in Vancouver, BC. The egg surface was decontaminated by spraying them with 70% ethanol. The EW and EY were separated and aseptically transferred into two sterile stomacher bags. The contents of each bag were homogenized for 40 s at 230 rpm. For sterility confirmation, both EW and EY were spread onto a TSA plate and incubated at 37 °C for 16–18 h. Aliquots of 8 mL of the homogenized liquid egg samples were transferred to 15 mL centrifuge tubes and inoculated with 1 mL of SE at a concentration of 10^6^ CFU/mL. The mixtures were vortexed at 3000 rpm to ensure even distribution of the bacterial cells in the viscous egg content. All samples were then incubated at 8 °C for 1 h to allow the bacteria to adapt to the liquid egg environment (Guenther et al., 2012).

Eight mL of the liquid egg samples, prepared as described above, containing 10^5^ CFU/mL of SE, were inoculated with 1 mL of 10^7^ PFU/mL of the two-phage cocktail (1:1 vol ratio). This resulted in a final MOI of 100. The same application was performed with a phage concentration of 10^8^ PFU/mL, resulting in a MOI of 1000. Control samples received 1 mL of BPW instead of phages. Three independent replicate experiments were conducted, and each measurement was performed in duplicate.

### 2.6 SE and phage enumeration in TSB

For SE enumeration at 0 h, the samples were enumerated after 15 min incubation at 8 °C to allow the bacterial population to accommodate to the incubation temperature. Aliquots of 100 μl from all samples were subsequently diluted with BPW and spread onto TSA in duplicate. The plates were incubated at 37 °C for 18 ± 2 h.

Phage titers were measured after 0 and 5 min, 1 and 6 h, and 1, 6, 12, 20, and 30 d using the double-layer agar overlay assay. Briefly, the samples in TSB were filtered using a 0.45 μm syringe filter to separate the supernatant and eliminate any unabsorbed phage particles. Then, 5 μl of the filtered samples were spotted in duplicate in serial dilutions on TSA plates already inoculated with the SE strain and grown for 16 h. Plaques were then counted after 18 ± 2 h incubation at 37 °C.

### 2.7 SE and phage enumeration in liquid eggs

SE enumeration was carried out at 0 and 6 h, and 1, 6, 12, 20 and 30 d. At 0 h, samples were enumerated after 15 min incubation at 8 °C to let the bacterial population adapt to the incubation temperature. Aliquots of 100 μL from all samples were subsequently diluted with BPW and spread onto TSA plates in duplicates, followed by incubation at 37 °C for 18 ± 2 h.

Phage titers were enumerated in the samples treated with a MOI of 100 using the same method described in section 2.2. Samples were collected after 0 and 5 min, 1 and 6 h, and 1, 6, 12, 20, and 30 d. To obtain phage lysate solution, liquid EW and EY samples were diluted 10-fold to reduce viscosity and passed through a 0.45 μm syringe filter to remove unabsorbed phages. A 5 μL aliquot of the filtered samples was spotted in duplicate onto TSA plates with SE as the host strain. Plaque enumerations were conducted after incubating the plates at 37 °C for 18 ± 2 h.

### 2.8 Phage stability assay in liquid eggs

Phage lysates were diluted in SM buffer to an initial concentration of approximately 10^8^ PFU/mL. An aliquot of 1 mL of the diluted phage lysate was inoculated into 9 mL of EW and EY, and the mixture was incubated at 8 °C for 30 d. Phage suspensions were serially diluted in SM buffer, and the effect of the liquid egg medium on phage titer was evaluated at 0 and 6 h, and 1, 6, 12, 20, and 30 d using the double-agar overlay method. The experiment was repeated three times for each phage treatment.

### 2.9 Statistical analysis

The experiments were independently performed in triplicate. A two-way ANOVA was conducted to examine the main effects and interactions of sampling day and phage treatment on each SE strain. Tukey's Honestly Significant Difference (HSD) post-hoc test was applied to all significant ANOVA results (α = 0.05). A one-way ANOVA was performed to determine the significance of the change in log differences in SE counts between the treated samples and untreated samples at different sampling time points (α = 0.05). A one-way ANOVA was also used to analyze the main effects of sampling day and SE strain on phage enumeration. For the phage stability assays, the final phage titer after 30 d of incubation was compared to the initial titer using a one-way ANOVA (α = 0.05) A P value of <0.05 was considered as statistically significant. All data analysis was performed using GraphPad Prism, version 9.5.1.

## 3 Results and Discussion

### 3.1 Initial assessment of phage cocktails infectivity

The composition of the two-phage cocktails was determined based on their ability to target specific SE serovars. In a previous study a heatmap was generated to assess the clearing patterns produced by *Salmonella* phages isolated from the BC environment (Brenner et al., 2020). The findings revealed that SI1, SS4, SF1, and SF2 could only infect SE strains, whereas SE15 and SE18 use *S.* Typhimurium as their isolation hosts and could also infect serovars including Brandenburg, Reading, Enteritidis, and Kentucky. Therefore, the inclusion of SE15 or SE18 in a phage cocktail can facilitate infection of different serotypes in practical applications. Phages SI1, SS4, SF1, and SF2 share the same isolation host and exhibit a high degree of genetic similarity, as they were previously classified into the same clusters on a dendrogram of whole-genome nucleotide alignment (Fong et al., 2019). This genetic similarity provides further support that these phages exclusively infect SE strains, possibly due to shared target mechanisms.

**Fig. S1:**
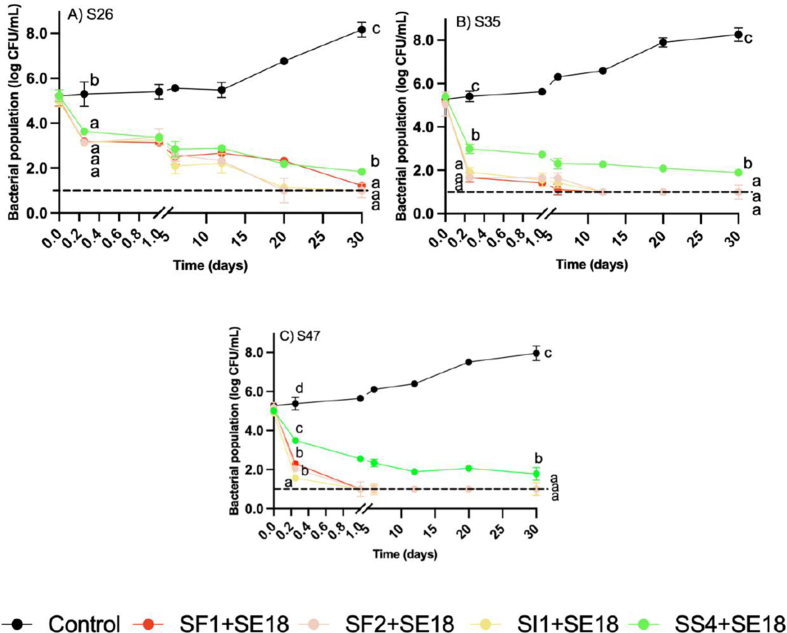
*In vitro* analysis of *S.* Enteritidis A) S26, B) S35, and C) S47 inhibition by six best formulated phage cocktails for 48 h at 25 °C. Data shown are the mean of three biological replicates ± SD.

In industrial applications it is common to employ cocktails consisting of 4 or 5 individual phages (Spricigo et al., 2013; Hungaro et al., 2013); however, 2-phage cocktails were assessed in this study due to longer inhibition times observed in comparison to 5-phage cocktails, offers economic and practical advantages. The antimicrobial efficacy of most of the phage cocktails was observed across all three SE strains, except two phage cocktails for S47 (Fig. S1). The lag phase of each SE strain was significantly extended by the six phage cocktails compared to treatment with individual phages. The average lag phase extension was 12 h for individual phages, whereas phage cocktails slowed bacterial growth up to 36 h.

For S26, cocktail SF2+SE18 exhibited the most suppression, with no regrowth observed until the end of the 48-h period. Most of other phage cocktails supressed growth for 24–42 h, and the maximum bacterial population after regrowth was lower than that of the control. The least effective cocktails were SF1+SE15 and SF2+SE15, which achieved a 2 h lag phase extension compared to the control. Similar patterns were observed for S35 and S47, where cocktails SI1+SE18, SF1+SE18, SF2+SE18, and SS4+SE18 displayed comparable inhibition times and significantly slower growth compared to the positive control and treatments with individual phages. SF1+SE15 and SF2+SE15 were the least effective against all three SE strains.

The performance of the phage cocktails can be attributed to synergistic interactions among phages, and the bacterial adaptations to counteract phage attacks such as changing or masking receptors, restriction-modification systems, or adaptive immunity through CRISPR/*Cas* systems (Labrie et al., 2010). The combination of multiple phages in cocktails targets bacteria in a complementary manner, delaying the emergence of bacterial resistance (Cai et al., 2018). By targeting different receptors on the bacterial surface, phage cocktails synergize and enhance the efficacy of infection, exerting selection pressure on the host bacteria and resulting in fitness costs and decreased frequencies of resistance (Simpson and Trent, 2019).

Although phage cocktails provide inhibition throughout a longer period compared to individual phages, SE populations were not completely eliminated in this study. This observation aligns with previous research in which the weakened inhibition of *Salmonella* growth was observed within 5 h of combined culture, indicating the emergence of phage resistance (Huang et al., 2018). Further analysis is necessary to determine whether the persistence of SE populations in this study was due to resistance development.

Four phage cocktails (SI1+SE18, SF1+SE18, SF2+SE18, and SS4+SE18) were selected for further experiments based on their effectiveness compared to the positive control and individual phage treatments by prolonging the lag phase and suppressing bacterial regrowth. The selection of these optimal phage cocktails provided a strong groundwork for assessing their efficacy in liquid eggs and investigating their practical potential as solutions to combat SE infections.

### 3.2 Single-step growth curves

**Fig. S2:**
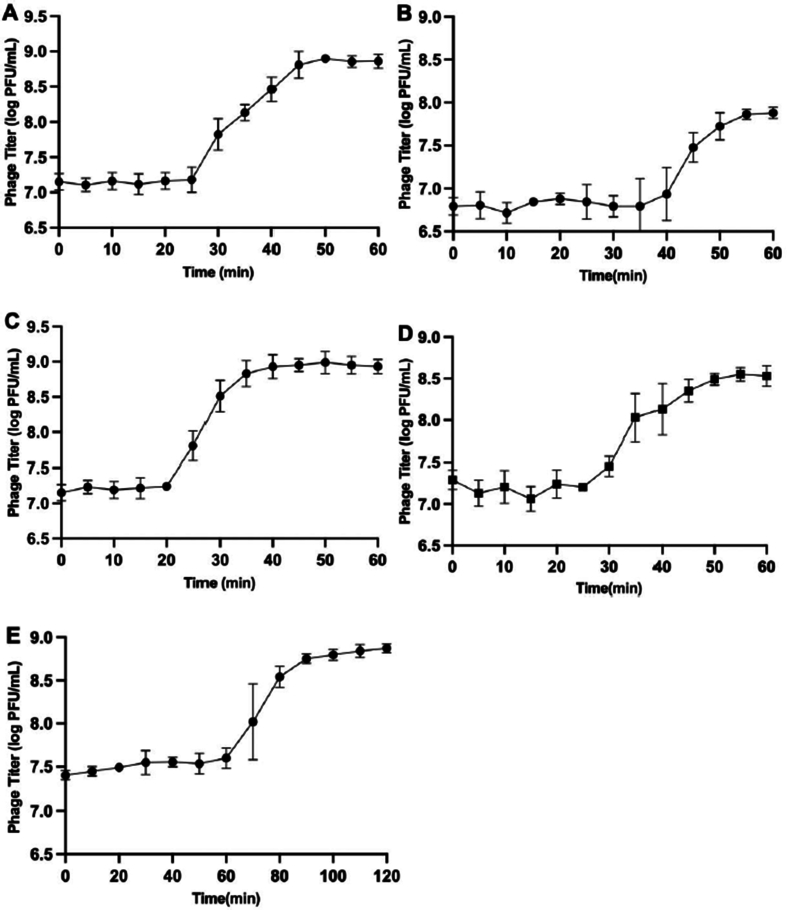
Single step growth curves of (A) SI1, (B) SS4, (C) SF1, (D) SF2, and (E) SE18. Data shown are the mean of three biological replicates ± SD.

Single-step growth curves were conducted to assess the infection potential of each phage, as illustrated in Fig. S2. The latent period represents the time taken for phage replication within host cells, and the burst size, indicates the number of phage progeny produced per host cell. SE18 exhibited the longest latent period of 60 min, while SI1, SF1, and SF2 showed approximately 25 min, and SS4 had 40 min. Regarding burst size, SI1 and SF1 produced 42 and 45 phages per cell, respectively, followed by SE18 with 35 phages per cell. SF2 and SS4 exhibited smaller burst sizes of 17 and 13 phages per cell, respectively.

A rapid latent period and a high burst size are indicative of greater potential for biocontrol (Gill and Hyman, 2010). Phages SI1 and SF1 exhibited a rapid latent period and a high burst size in this study, suggesting their effective control of SE populations. Conversely, phage SE18 exhibited a longer latent period, possibly due to its extended replication time within the host, resulting in the production of more progeny (Abedon, 2009). In contrast, phage SS4 exhibited a longer latent period and a smaller burst size, indicating potential limitations as a standalone biocontrol agent. However, treatments with SS4+SE18 showed a synergistic inhibitory effect in the *in-vitro* infectivity assay, indicating that the combination of phages may enhance their collective biocontrol capabilities. Other studies found that *Myoviridae* phages infecting *Salmonella* typically exhibit burst sizes ranging from 150 to 250 phages per cell, with a latent period of approximately 20 min (Carey-Smith et al., 2006; Duc et al., 2018). The difference in the findings of this study may be due to experimental conditions such as temperature and host concentration, which significantly impact infection parameters.

### 3.3 Reduction of SE by phage cocktails in TSA over a 30-day period

**Fig. S3:**
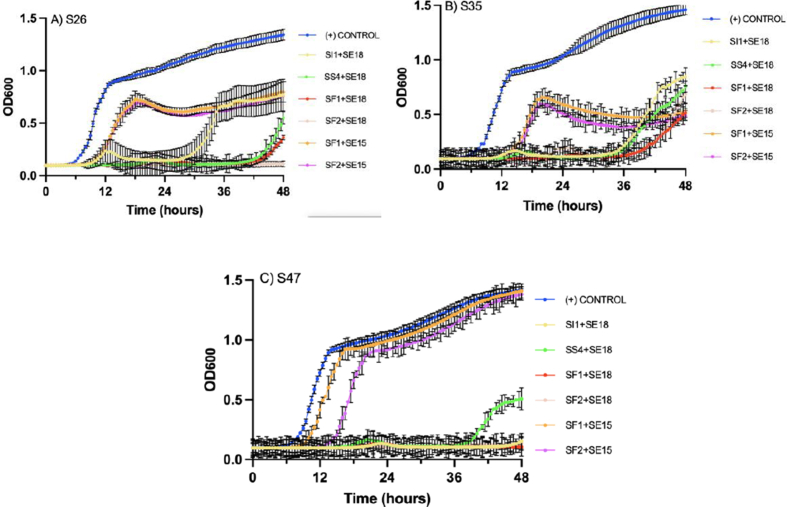
Population of SE strain A) S26, B) S35, and C) S47 over an experimental timeframe of 30 days with and without phage treatments. Different colors represent different treatments, including phages SI1+SE18, SS4+SE18, SF1+SE18 and SF2+SE18. Data shown are the mean of three biological replicates ± SD. Lowercase letters indicate statistically significant differences (P < 0.05).

Populations of SE strains S26, S35, and S47 were assessed at different time points (0 and 6 h, and 1, 6, 12, 20, and 30 d) (Fig. S3). The reduction in S26 was dependent on sampling day (P < 0.0001), and there was an interaction between sampling day and phage treatment (P < 0.0001). The positive control without phage treatment showed a significant increase to 8 log CFU/mL after day 12 (P < 0.001) and remained at that level until the end of day 30 at 8 °C (Fig. S3A). All four phage treatments exhibited a significant 2 log decrease at 6 h (P < 0.001) and nearly eliminated S26 to below the LOD, <10 CFU/mL by the end of day 30. No significant differences were observed between phage treatments across all time points (P > 0.05), except that SS4+SE18 exhibited a lower reduction compared to the others.

Similarly, all four phage treatments resulted in significant decreases in S35 populations (P < 0.0001) (Fig. S3B). At 6 h, SI1+SE18, SF1+SE18, and SF2+SE18 achieved reductions of 3.5 log CFU/mL, whereas SS4+SE18 showed a significantly lower 2-log reduction (P < 0.0001). SI1+SE18, SF1+SE18, and SF2+SE18 reduced S35 to below the LOD by day 12 until day 30; however, SS4+SE18, reduced S35 to 2 log within the same time frame. Treatments with SI1+SE18, SF1+SE18, and SF2+SE18 were equally effective (P > 0.05), while SS4+SE18 exhibited significant differences at day 30 (P < 0.0001); however, all four phage treatments were significantly different from the positive control at day 30 (P < 0.001).

The reduction of S47 was dependent on sampling day (P < 0.0001), and there was an interaction between sampling day and phage treatment (P < 0.0001). The positive control showed a gradual increase to 8 log CFU/mL by the end of day 30 (Fig. S3C). Phage treatments exhibited similar reductions observed with S35. At 6 h, SI1+SE18, SF1+SE18, and SF2+SE18 reduced the S35 population by 3.5 log CFU/mL, while SS4+SE18 was less effective, with a reduction of 1.5 log at 6 h. All phages were equally effective in reducing S47 (P > 0.05), except for SS4+SE18, which was less effective.

The results demonstrated the strong lytic activity of the selected phage cocktails, as all four formulations effectively reduced each SE strain within 6 h (Fig. S3). SE populations continued to decrease throughout the experiment, reaching less than the LOD by day 30. The chosen MOI of 100, representing the ratio of phage particles to bacterial cells, was appropriate for achieving this level of efficacy in a liquid medium, which aligns with previous studies that demonstrated effectiveness of an MOI of 100 in reducing bacterial populations (Fong et al., 2019; Ly-Chatain, 2014; Yi et al., 2021; Zhang et al., 2021). The MOI can influence the speed at which phages reduce bacteria. For example, a study by Azari et al. (2023) demonstrated that at 4 °C, SE populations were below the LOD after 3 and 6 h at MOIs of 10,000 and 10, respectively. Among the three SE strains tested, phage cocktails displayed the highest effectiveness against S47. Three of the four cocktails reduced its population below the LOD by day 1, indicating that S47 may be more susceptible to phage treatments than the other strains. Further investigation into the specific characteristics and virulence factors of S47 could provide insights into its susceptibility to phages.

In the absence of phages, all three SE strains demonstrated survival and growth after day 12 at 8 °C. This could be attributed to the time required for bacterial populations to adapt to the low temperature through the expression of cold stress proteins, such as RpoS (Ricke et al., 2018). *Salmonella* populations have been reported to multiply to high levels (10^5^–10^8^ CFU/mL or g) at lower temperatures in various types of food products (Guenther et al., 2012).

### 3.4 Fate of SE in liquid egg white treated with phage cocktails

**Fig. 1:**
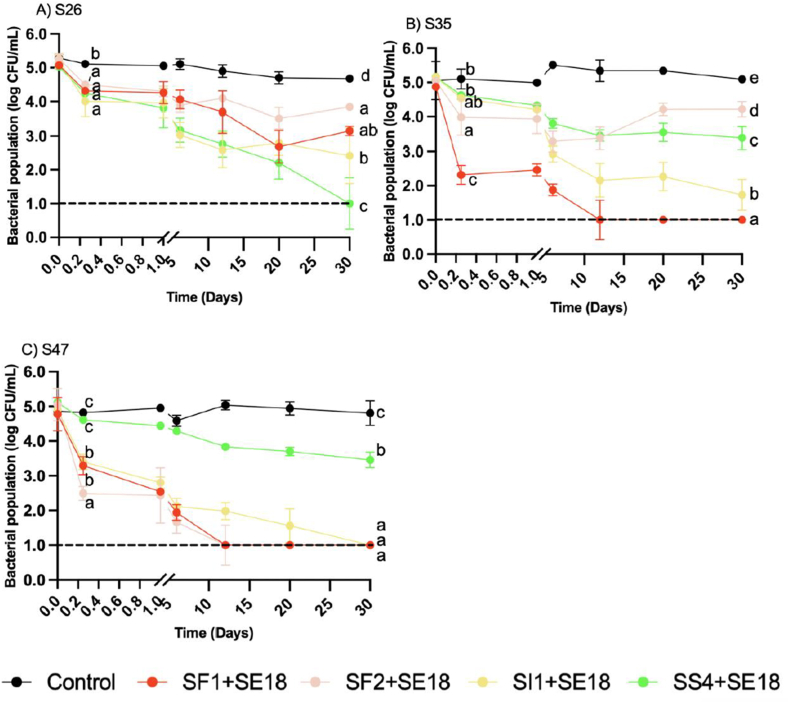
Bacterial population of SE strains A) S26, B) S35, and C) S47 in egg white over 30 days with phage treatments under MOI 1000. Different colors represent different phage treatments, including phage cocktails SI1+SE18, SS4+SE18, SF1+SE18 and SF2+SE18. Data shown are the mean of three biological replicates ± SD. Lowercase letters indicate statistically significant differences (P < 0.05).

**Fig. 2:**
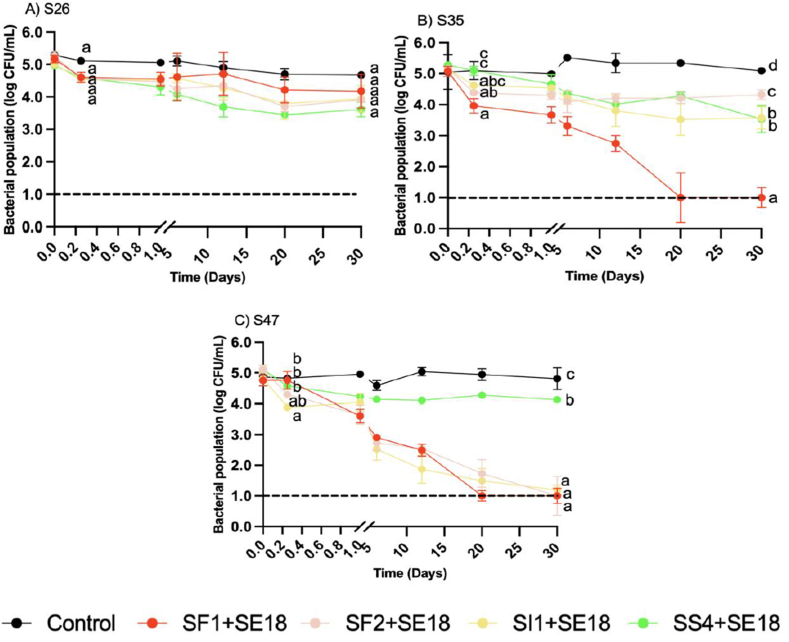
Bacterial population of SE strains A) S26, B) S35, and C) S47 in egg white over 30 days with phage treatments under MOI 100. Different colors represent different phage treatments, including phage cocktails SI1+SE18, SS4+SE18, SF1+SE18 and SF2+SE18. Data shown are the mean of three biological replicates ± SD. Lowercase letters indicate statistically significant differences (P < 0.05).

The enumeration of three SE strains in EW was carried out at various time points (0 and 6 h, and 1, 6, 12, 20, and 30 d) with the treatment of four different phage cocktails at MOIs of 1000 and 100 (Fig. 1 & Fig. 2). The reduction of all SE strains was dependent on sampling day (P < 0.001) and phage treatment (P < 0.001), and there was a significant interaction between sampling day and phage treatment (P < 0.001). All phage cocktails demonstrated reduction of each SE strain at both MOIs.

At MOI 1000, all phage treatments significantly reduced S26 by 1 log at 6 h (P < 0.05) (Fig. 1). SS4+SE18 was the most effective, with consistent reductions below the LOD by day 30, which significantly differed from the other cocktails (P < 0.0001). At MOI 100, all cocktails achieved a 0.5 log reduction at 6 h with no significant differences between them or the positive control (P > 0.05). By day 30, all phage cocktails achieved a ∼1 log reduction of S26 with no significant differences from the positive control (P > 0.05) (Fig. 2).

At MOI 1000, SF1+SE18 and SF2+SE18 significantly reduced S35 at 6 h (P < 0.0001). SF1+SE18 was the most effective, reducing counts from 5 log to 2 log (P < 0.001) (Fig. 1A). By day 12, SF1+SE18 remained the most effective, with counts below the LOD, while SI1+SE18 and SS4+SE18 achieved reductions from 5 log to 2 log and 3.5 log, respectively. In contrast, treatments with SF2+SE18 were not effective, with regrowth of S35 over time, and an insignificant reduction at day 30 compared to day 0. At MOI 100, significant reductions of S35 were achieved with all four phage cocktails (P < 0.001) compared to the positive control at day 30 (Fig. 2). SF1+SE18 achieved reductions below the LOD, but at a slower rate with MOI 100 than MOI 1000. All phage treatments at both MOIs significantly reduced S35 compared to the positive control at day 30 (P < 0.001).

Similar trends were observed with S47 at MOI 1000. SF1+SE18 was the most effective, reducing S47 below the LOD by day 12. SF2+SE18 and SI1+SE18 exhibited effective reduction, with SI1+SE18 achieving reductions below the LOD by day 30. SS4+SE18 treatment achieved significantly less reduction compared to the other phage cocktails at all time points (P < 0.001). At MOI 100, all phage cocktails followed the same trend as at MOI 1000, but with weaker efficacy in reducing the bacterial population (Fig. 2). Notably, SI1+SE18, SF2+SE18, and SF1+SE18 still achieved reductions below the LOD after 30 d, with SF1+SE18 achieving reductions below the LOD by day 20. SS4+SE18 achieved a significant reduction (P < 0.05) compared to the positive control at day 30, but it was less effective than the other cocktails at both MOIs.

The results indicated that phage treatments in liquid EW can consistently achieve SE reductions of 3–4 log CFU/mL after 30 d. Similar reductions have been observed in other food matrices, such as turkey deli meat, chocolate milk, hot dogs, and seafood, when treated with phages at an MOI of 10^5^ at 8 °C (Guenther et al., 2012); however, regrowth was observed over time. Similarly, Spricigo et al. (2013) found that chicken breasts dipped in a phage cocktail solution at an MOI of 1000 for 5 min produced a reduction of 0.9 log CFU/g over a 7-d period at 4 °C. In the current study, a larger reduction was achieved in liquid EW, suggesting that the application of phages in this matrix was highly effective, potentially due to the presence of antimicrobial compounds in EW, such as lysozyme, egg transferrin, and other bacteriostatic substances, which may work synergistically with phage treatments (Kovacs-Nolan et al., 2005).

### 3.5 Fate of SE in liquid egg yolk treated with phage cocktails

**Fig. 3:**
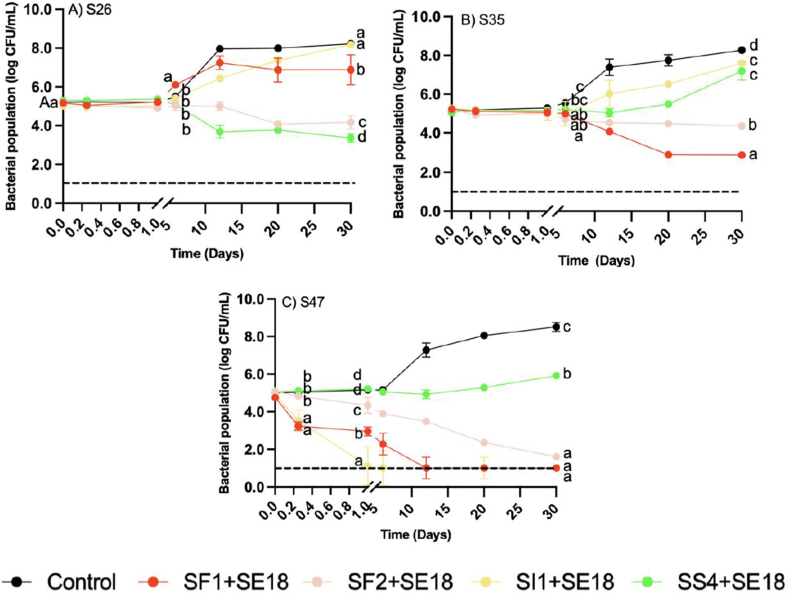
Bacterial population of SE strains A) S26, B) S35, and C) S47 in egg yolk over 30 days with phage treatments under MOI 1000. Different colors represent different phage treatments, including phage cocktails SI1+SE18, SS4+SE18, SF1+SE18 and SF2+SE18. Data shown are the mean of three biological replicates ± SD. Lowercase letters indicate statistically significant differences (P < 0.05).

The overall trend of phage efficacy in EY was different from that observed in EW, as regrowth of all three SE strains was observed for certain phage treatments at both MOIs. The positive control for EY exhibited a significant increase of 3 log by day 12 (P < 0.001), which was sustained until the end of day 30 (Fig. 3). This suggested that the EY growth medium provided a conducive environment for the proliferation of bacteria, leading to a substantial population increase over time (Gantois et al., 2009). Nonetheless, regrowth in phage-treated EY took longer to reach the maximal concentration compared to that of the positive control, indicating that the phage treatment slowed regrowth to some extent.

**Fig. 4:**
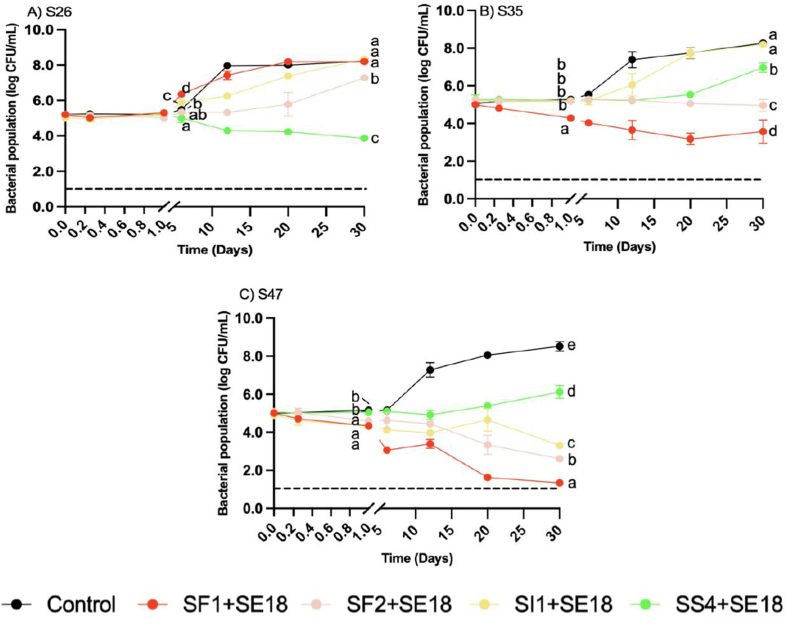
Bacterial population of *S.* Enteritidis strains A) S26, B) S35, and C) S47 in egg yolk over 30 days with phage treatments under MOI 100. Different colors represent different phage treatments, including phage cocktails SI1+SE18, SS4+SE18, SF1+SE18 and SF2+SE18. Data shown are the mean of three biological replicates ± SD. Lowercase letters indicate statistically significant differences (P < 0.05).

At both MOIs, no changes in S26 populations were observed until day 6 when treated with all four phage cocktails (Figs. 3 and 4). At MOI 1000, significant growth was observed at day 6 after the SF1+SE18 treatment (P < 0.05). The bacterial population continued to increase and reached 7 log CFU/mL after 30 d, which was comparable to the positive control. A similar trend was observed with SI1+SE18 at MOI 1000. At MOI 1000, both SS4+SE18 and SF2+SE18 were more effective compared to SI1+SE18 and SF1+SE18 with more significant reductions of S26 at day 30 compared to the positive control (P < 0.001). At MOI 100, the same pattern was observed for each phage cocktails, and the effect of phages started to be seen after day 6.

At MOI 1000, SI1+SE18 resulted in a 2-log increase of S35 by day 30. Despite its efficacy at reducing S26, SS4+SE18 resulted in an increase in S35 at day 30. SF1+SE18 and SF2+SE18 demonstrated significant reductions by day 30 (P < 0.001), although SF2 reductions were less than SF1. At MOI 100, the bacterial population after 30 d did not differ from that of MOI 1000 for all phage cocktails, and the trends in reduction were similar to those observed at MOI 1000.

At MOI 1000, SI1+SE18 was the most effective against S47 with reductions below the LOD on day 1 (Fig. 3). SI1+SE18 exhibited insignificant differences with SF1 and SF2 on day 30 (P > 0.05), as the S47 population was at the LOD. However, SS4+SE18 showed gradually increasing S47 populations. S47 populations were significantly lower for all phage cocktails compared to the positive control after 30 d (P < 0.001). At MOI 100, similar patterns were observed, although with lesser magnitudes of reduction.

The results of this study demonstrated the challenges and limitations associated with the application of phage cocktails in SE-contaminated liquid EY. Other studies with phage treatments in EY obtained similar results. For example, a 2.6 log CFU/mL reduction of *S*. Typhimurium in EY was observed following phage FO1-E2 treatment with MOI 10^5^ after 2 d at 8 °C (Guenther et al., 2012). Decreased reductions in EY might be due to reduced diffusion and inhomogeneous distribution of phage particles in the highly viscous EY matrix, or from other compounds limiting the accessibility of phages to host bacteria, impacting phage adsorption and infection (Gnanou Besse et al., 2005).

The regrowth observed in some phage treatments highlighted the difficulty in achieving complete elimination of SE in this matrix. Regrowth may be attributed to the immobilization of virus particles in the EY due to its high viscosity, thus preventing phage diffusion. This may have enabled the replication of SE in protected niches, while the EY provided nutrients beneficial to bacterial proliferation (Spricigo et al., 2013). A similar regrowth trend was observed by Guenther et al. (2012), as phage-treated EY samples demonstrated regrowth after 6 d that was comparable to the positive control. Additionally, phages in refrigerated food samples became undetectable after 2 weeks; therefore, the prolonged incubation time may have permitted regrowth of the targeted bacteria (Hong et al., 2016).

Overall, MOI 1000 produced more substantial and rapid reductions than MOI 100 in EW and EY. Similarly, Guenther et al. (2012) reported that higher MOI phage treatments produced more significant reductions of *Salmonella* below the LOD at 8 °C in liquid egg. However, Zhang et al. (2021), reported that higher MOIs achieved faster reduction, but resulted in more rapid development of bacterial resistance. Therefore, careful consideration should be given to selecting an appropriate MOI that achieves the most effective reduction while delaying bacterial resistance and regrowth following phage treatment.

Considering the challenges and limitations observed in the application of phage cocktails to SE-contaminated liquid EY, it is worth exploring the combination of phage treatment with other preservation methods, such as pasteurization, pulsed light, or gamma radiation (Yi et al., 2021). For example, combining phage treatment with heat resulted in higher *Salmonella* reductions, as the lytic effect from the phage disrupts the integrity of the bacterial cell envelope, thereby increasing the susceptibility of cells to other stress such as heat (Wu, 2008).

In practical applications, the level of *Salmonella* contamination is typically lower than in laboratory settings. A USDA risk assessment reported that contaminated eggs harbor 1–100 bacterial cells per egg (U.S. Department of Agriculture, FSIS, 2005). Furthermore, only 15% of all contaminated eggs experienced >1 log growth of *Salmonella* during storage (U.S. Department of Agriculture, FSIS, 2005). Therefore, the addition of phages at the levels tested in this study may be more effective against *Salmonella* in real-world scenarios.

Despite these challenges, the observations of delayed regrowth in phage-treated EY indicated that the phage treatments still offer an inhibitory effect. It is important to optimize the phage treatment conditions by selecting appropriate phages, determining the optimal MOI, and ensuring an even distribution of the phage inoculum to facilitate effective diffusion of virus particles for the maximum reduction while minimizing regrowth and the potential for bacterial resistance development (Guenther et al., 2009).

### 3.6 Changes in phage titers during the infection of SE over a 30-day period

**Fig. 5:**
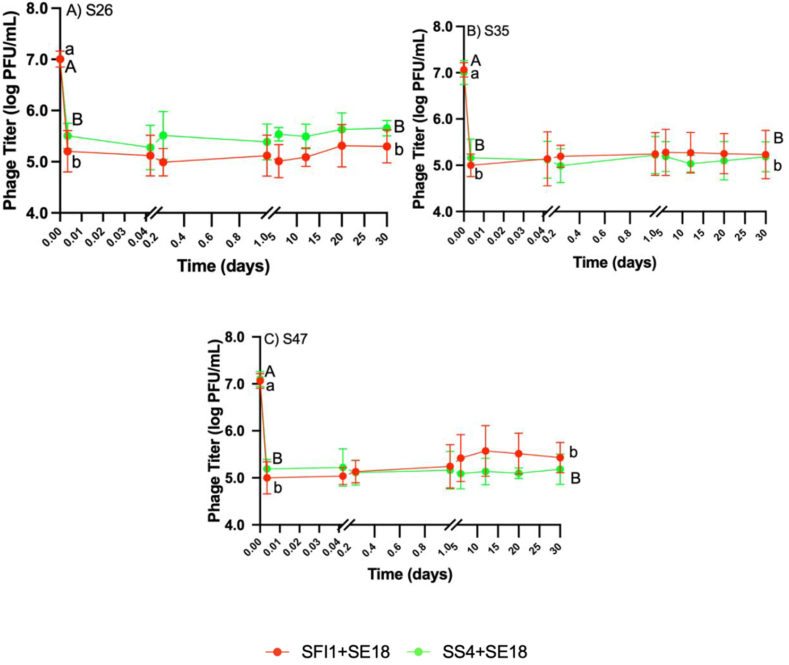
Phage titer recovery in TSB when tested against SE strain A) S26, B) S35, and C) S47 at 8 °C. Error bars represented in means and standard deviations. Different letters (a–b) denote statistically significant differences (P < 0.05) between timepoints for phage cocktail SF1+SE18. Different letters (A–B), obtained using Tukey's honestly significant difference test (HSD), denote statistically significant differences (P < 0.05) between timepoints for phage cocktail SS4+SE18.

The titers of two phage cocktails (SF1+SE18 and SS4+SE18) were enumerated in TSB against the three SE strains over 30 d at 8 °C (Fig. 5). The rationale behind the selection of phage combinations SF1+SE18 and SS4+SE18 for titer assessment was because they demonstrated relatively different reduction patterns for all three SE strains.

SF1+SE18 and SS4+SE18 showed a rapid initial decrease followed by a leveling off over time across all SE strains (Fig. 5). The decrease in phage concentration was accompanied by a reduction in SE population, indicating their interdependence, as phages rely on active replication within host cells (Hong et al., 2016).

The trend for phage titers observed for all SE strains over 30 d was nearly identical. Phage titers of each strain exhibited a 2-log reduction within the first 5 min (P < 0.001), which was consistent with previous studies on high MOI phage behavior (Li et al., 2020; Wang et al., 2017). The rapid initial decrease in phage titers can be attributed to the higher concentration of phages adhering to the bacterial cell surface, leading to cell envelope damage and subsequent cell death without the need for phage multiplication (Abedon, 2012; Duc et al., 2020; Sonalika et al., 2020). It would be beneficial for future experiments to confirm this lysis from without phenomenon by accurately measuring the timing of lysis using a single-step growth curve of phages specifically at 8 °C (Abedon, 2012) or using spot testing methods (Kutter, 2009; Fong et al., 2017).

Phage cocktail treatments at MOI 100 in this study demonstrated this phenomenon, as there was no significant increase in phage populations despite a substantial reduction of SE. Conversely, lysis from within in phage treatments at lower MOIs likely depended on the full replication cycle within bacterial cells for lysis to occur (Abedon, 2012). The persistence of phage titers up to day 30 can be attributed to the low metabolic activity of bacteria at the low incubation temperature, as phages require active bacterial replication to produce phage progeny, and the lysis from within process is hindered under such conditions (Park et al., 2020).

**Fig. 6:**
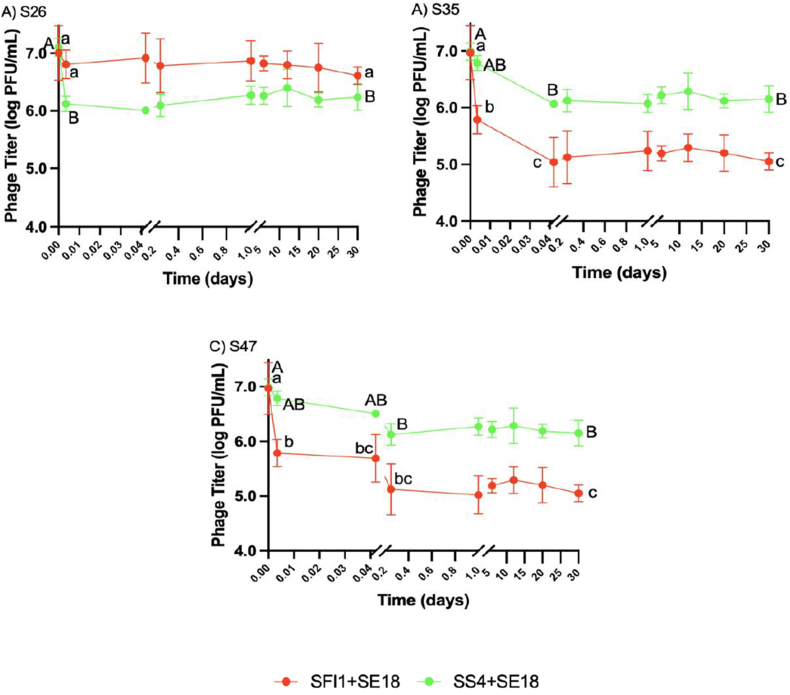
Phage titer recovery in egg white when tested against SE strain A) S26, B) S35, and C) S47. Error bars represented in means and standard deviations. Different letters (a–c) denote statistically significant differences (P < 0.05) between timepoints for phage cocktail SF1+SE18. Different letters (A–B) denote statistically significant differences (P < 0.05) between timepoints for phage cocktail SS4+SE18.

**Fig. 7:**
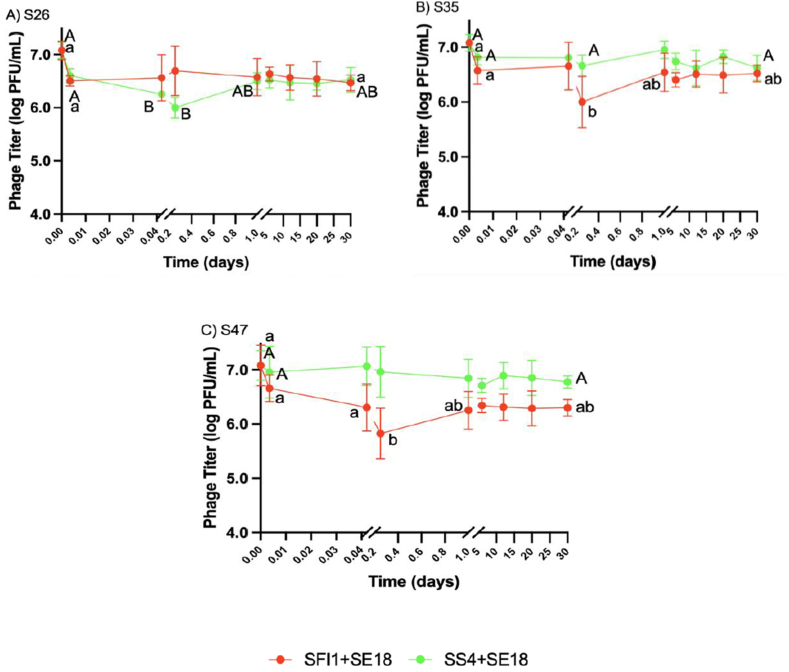
Phage titer recovery in egg yok when tested against SE strain A) S26, B) S35, and C) S47. Error bars represented in means and standard deviations. Different letters (a–b) denote statistically significant differences (P < 0.05) between timepoints for phage cocktail SF1+SE18. Different letters (A–B) denote statistically significant differences (P < 0.05) between timepoints for phage cocktail SS4+SE18.

Phage titers of SF1+SE18 and SS4+SE18 were measured at various time points (0 and 5 min, 1 and 6 h, and 1, 6, 12, 20, and 30 d) in EW (Fig. 6) and EY (Fig. 7). In all strains, phage concentrations decreased, indicating attachment to the bacterial cell membrane. SF1+SE18 and SS4+SE18 were selected for titer measurements because they exhibited the most diverse reduction patterns for most SE strains in EW and EY samples.

In EW with S26, SF1+SE18 showed an insignificant reduction at 5 min and maintained a constant titer throughout the 30-d period (P > 0.05). On the other hand, SS4+SE18 exhibited a significant reduction (P < 0.05) at 5 min and then remained constant until day 30. The different reduction patterns in the two cocktails can be attributed to the bacterial killing curve in EW, where S26 was predominantly reduced by cocktail SS4+SE18 while SF1+SE18 was not effective. In S35 and S47, SF1+SE18 exhibited a significant reduction in the first 5 min and SS4+SE18 showed an insignificant reduction. Titers of both phage cocktails remained constant thereafter. This suggests that the phages attached to the bacteria more effectively when there was a higher reduction in bacterial population. The absence of a phage burst can be explained by the lysis from without mechanism (Sonalika et al., 2020). This is supported by the observed decrease in phage titer accompanied by a decrease in SE population, indicating the absence of phage progeny release.

In EY, a decreasing trend in phage titer was also observed, but the magnitude of the initial 5 min decrease was less than in EW and TSB. Similarly, titers of SS4+SE18 decreased more in S26 due to greater efficacy of the phage cocktail, whereas the titers of SF1+SE18 decreased more in S35 and S47 due to a larger reduction of the bacterial population compared to SS4+SE18. From 6 h to 1 d, a non-significant increase in phage titer was observed across all three SE strains. Subsequently, the titers of all three phage cocktails remained constant throughout the 30-d period with no significant changes (P > 0.05). These findings align with previous research in which phage titers remained stable over a 6-d period in various foods (Guenther et al., 2012). The less significant decrease in phage titer observed in the first 5 min may be due to the high viscosity. Guenther et al. (2012) proposed a similar theory, suggesting that the high viscosity of liquid food immobilizes virus particles, thus limiting phage infection of *Salmonella* beyond the initial decrease.

In summary, the initial decrease in phage titers varied between SF1+SE18 and SS4+SE18 in EW and EY, with a less pronounced decrease observed in EY due to its higher viscosity. These observations, coupled with the absence of a phage burst, suggested the potential involvement of a lysis from without mechanism, highlighting its relevance in understanding phage activity. These findings emphasized the importance of considering the early stages of phage treatment and the influence of viscosity when aiming to reduce bacterial contamination in liquid egg products.

**Fig. 8:**
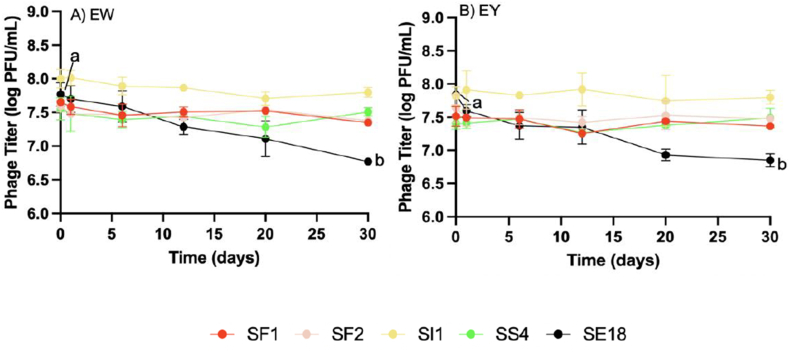
Stability of phage SF1, SF2, SI1, SF2 and SE18 in A) egg white and B) egg yolk over a period of 30 days at 8 °C. Data shown are the mean of three replicates ± SD. Different letters denote statistically significant differences (P < 0.05).

Individual phage titers were evaluated over a 30-d incubation period at 8 °C in EW and EY, without bacterial inoculation, to assess their stability in egg storage conditions. In EW, all phage concentrations remained constant after 30 d, except for SE18, which exhibited a 1 log reduction (P < 0.001) (Fig. 8A). Similar trends were observed in EY, with SE18 showing a 1 log reduction while the concentrations of the other four phages remained stable (Fig. 8B). This stability in EW can be attributed to the resilience of *Siphoviridae* phages, such as SI1, SF1, and SS4 (Fong et al., 2017). A previous study indicated that *Siphoviridae* phages can withstand adverse temperature and pH conditions (Lasobras et al., 1997). Consequently, these phages can tolerate the alkaline pH of EW, ranging from 7.6 to 9.2 during storage (Zhang et al., 2021).

However, SE18 demonstrated instability within the food matrix. Research has shown that phage stability in food systems is complex and can be influenced by various compounds present in the food (García-Anaya et al., 2020). For example, *Salmonella* phage SJ2 demonstrated long-term stability over 90 d at 8 °C in cheese samples (Modi et al., 2001). In contrast, the titers of two *Listeria monocytogenes* phages declined to undetectable levels within 30 min in apple slices due to the acidic nature of the environment (Leverentz et al., 2003). Despite the limited information regarding phage stability in specific food compounds, previous studies have reported differential phage response due to pH, temperature, food matrix properties, and the presence of antiphage compounds (Jończyk-Matysiak et al., 2019).

Interactions between phages and food compounds can lead to the formation of phage-compound complexes. For example, fats and proteins in milk have been associated with antiphage activity from the formation of casein-phage complexes (Das and Marshall, 1967). Unsaturated fatty acids of C18 and C20 length also impacted *E. coli* phage T5 stability (Hirotani et al., 1991). In this study, the instability of SE18 in EW and EY can be attributed to the presence of proteins and fats, which may act as antiphage compounds. The formation of phage-protein or phage-fat complexes may have contributed to the observed instability in the egg medium. Furthermore, the increasing pH of egg over time, up to 12, could also reduce the stability of SE18 in EW. This finding is supported by a study that reported the instability of *Salmonella* phage at pH levels between 10 and 12 (Ahiwale et al., 2013).

The complexity of phage stability in food matrices highlighted the need to understand factors influencing phage behavior in different food environments. The findings from this study provided valuable insights into the interactions between phages and food components, which is essential for developing effective phage-based strategies in liquid egg products. Further research is required to identify specific food components responsible for phage instability and optimize phage formulations to enhance their efficacy in food applications.

## 4 Conclusion

The efficacy of the phage cocktails against SE strains in EW and EY at MOIs of 100 and 1000 at 8 °C over 30-d was demonstrated. The effectiveness varied across SE strain and was influenced by MOI and medium composition. SE reductions ranged from 1 to over 4 log CFU/mL, with MOI 1000 consistently achieving higher reductions, particularly at 8 °C. The challenge of reducing bacterial populations in EY highlights the need to optimize phage treatment conditions and understand the impact of egg components on phage performance. Additionally, the stability of phages in EW and EY without bacterial inoculation at 8 °C were observed, except for SE18, which showed slightly reduced activity. Furthermore, the decrease in phage titer in EW might be related to the stronger attachment in EW, which is likely due to its less viscous nature compared to EY. The decrease in phage titers raises a key challenge in utilizing phages for pathogen control in foods, specifically in terms of maintaining optimal phage titers to inhibit bacterial growth over extended periods of time. Replenishing phage titers through repeated applications may be a potential solution to delay the regrowth of *Salmonella* throughout long-term storage.

In summary, this study highlights the potential of phage cocktails for controlling *Salmonella* in liquid environments such as TSB and liquid eggs. Further research is required to elucidate the mechanisms of bacterial lysis, optimize treatment conditions, and explore the impact of egg components on phage performance.

## FUNDING

Funding for this project has been provided by the Governments of Canada and British Columbia through the Canadian Agricultural Partnership, a federal-provincial-territorial initiative. The program is delivered by the Investment Agriculture Foundation of BC (INV 133).

## CRediT authorship contribution statement

**Jiangning He:** Methodology, Investigation, Visualization, Writing – original draft. **Catherine W.Y. Wong:** Methodology, Writing – original draft. **Danielle M. Schultze:** Methodology, Writing – review & editing. **Siyun Wang:** Supervision, Conceptualization, Writing – review & editing, Funding acquisition.

## Declaration of competing interest

The authors declare that they have no known competing financial interests or personal relationships that could have appeared to influence the work reported in this paper.

## Data Availability

Data will be made available on request.
